# Association between weight-adjusted waist circumference index and risk of cognitive decline in Chinese hypertensive patients: a case-control study

**DOI:** 10.3389/fpubh.2025.1632358

**Published:** 2025-07-16

**Authors:** Mengdi Zhang, Donghai Liu, Xipeng Yan, Zhenchao Niu, Yudong Zhang, Lei He, Huachen Jiao

**Affiliations:** ^1^Department of First Clinical Medical College, Shandong University of Traditional Chinese Medicine, Jinan, Shandong, China; ^2^School of Laboratory Animal & Shandong Laboratory Animal Center, Shandong First Medical University & Shandong Academy of Medical Sciences, Jinan, China; ^3^Department of Traditional Chinese Medicine, Jining First People's Hospital, Jining, Shandong, China; ^4^Department of Cardiology, Affiliated Hospital of Shandong University of Traditional Chinese Medicine, Jinan, Shandong, China; ^5^Peripheral Vascular Department, Affiliated Hospital of Shandong University of Traditional Chinese Medicine, Jinan, Shandong, China; ^6^Department of Cardiology, Qufu Hospital of Traditional Chinese Medicine, Jining, Shandong, China

**Keywords:** weight-adjusted-waist index, obesity, hypertension, cognitive decline, risk factors

## Abstract

**Background:**

As a new obesity-related index, the weight-adjusted waist circumference index (WWI) seems to be a good predictor of cognitive decline in hypertensive patients. This study aimed to verify the relationship between WWI and cognitive decline in Chinese hypertensive patients.

**Methods:**

Data were obtained from the Hypertension Follow-up System of Shandong Province. WWI was calculated by dividing waist circumference by the square root of body weight, and cognitive decline was judged based on Mini-Mental State Examination (MMSE) scale scores. Multivariate logistic regression models and subgroup analyses were used to assess the relationship between WWI and cognitive decline in hypertensive patients.

**Results:**

A total of 2,204 participants were included. There was a positive association between WWI and cognitive decline in hypertension (HCD). After adjusting for all covariates, a one-unit increase in WWI was associated with a 12% increase in the risk of cognitive decline in hypertensive patients (OR: 1.12, 95%CI: 1.04–1.21). In addition, the multivariable-adjusted OR for the highest quartile (11.16–16.76) compared with the lowest quartile of WWI (7.00–10.11) was 1.61 (95% CI: 1.30–2.00).

**Conclusion:**

In Chinese hypertensive patients, high levels of WWI were significantly associated with an increased risk of cognitive decline. This finding suggests that WWI may be an indicator of the risk of cognitive decline affecting hypertensive patients.

## Introduction

The relationship between hypertension and cognitive decline is garnering increasing attention. Hypertension is not only a significant risk factor for cardiovascular disease but may also contribute to cognitive deterioration. Research has demonstrated that hypertensive patients face a markedly elevated risk of cognitive decline attributable to various etiologies, encompassing vascular dementia and Alzheimer’s disease (1–3). In a study involving women, those with hypertension who did not effectively manage their blood pressure exhibited a 30% higher risk of cognitive decline compared to untreated patients with well-controlled blood pressure (4).

The social burden of hypertension accompanied by cognitive decline is significant and cannot be ignored. As ages, cognitive decline not only diminishes the quality of life for patients (5), but also places significant strain on families and the broader community (6). Moreover, hypertension associated with cognitive decline is recognized as a critical factor influencing all-cause mortality among the older adults. Studies have shown that patients with both cognitive impairment and hypertension have an increased risk of all-cause death compared to patients with only cognitive impairment or hypertension (7). Consequently, there is an urgent need to identify a variable and measurable indicator that can help reduce the incidence of cognitive decline in individuals with hypertension.

Park et al. proposed the weight-adjusted waist circumference index (WWI) as a novel measurement in 2018 (8, 9). Unlike the body mass index (BMI) or waist circumference (WC), WWI considers the relationship between waist circumference and body weight, offering a more comprehensive understanding of body fat distribution (10). WWI was calculated as WC (cm) divided by the square root of weight (kg) (9), because the calculation of WWI only needs to measure waist circumference and weight, and the data is easy to obtain, it is suitable for large-scale epidemiological investigation and clinical screening. Recent studies have indicated that WWI may be linked to the development of hypertension (8, 11) and dementia (12).

Therefore, the aim of this study was to evaluate the association between WWI and cognitive decline in a hypertensive population in China, thereby providing a foundation for the early identification of cognitive decline among hypertensive patients.

## Methods

### Study design and data sources

This study was a multicenter observational investigation involving hospitalized patients with hypertension. The Affiliated Hospital of Shandong University of Traditional Chinese Medicine collaborated with nine hospitals located in Jinan, Weifang, Yantai, Tai’an, Dongying, and Jining. Hypertensive patients underwent comprehensive evaluation of: (a) general health status, (b) physical activity levels, (c) sleep quality, and (d) cognitive function. A total of 6,098 patients were enrolled from May 2022 to July 2024. The subjects of this study were patients with essential hypertension aged over 40 years. Patients with secondary hypertension, renal impairment, comorbid mental disorders, and those with a history of alcohol or psychotropic drug abuse were excluded. After matching patients with hypertension and cognitive decline to cognitively normal hypertensive patients by age and sex in a 1:1 ratio, 2,726 patients were included in the final analysis, comprising 1,363 patients with cognitive decline and 1,363 cognitively normal patients.

### Sample size calculation


$$\frac{{\left[\frac{u_{\alpha }}{2}+u_{\beta }\sqrt{\frac{\text{OR}}{1+\text{OR}}(1-\frac{\text{OR}}{1+\text{OR}})}\right]}^{2}/{\left(\frac{\text{OR}}{1+\text{OR}}-0.5\right)}^{2}}{\left(p_{0}q_{1}+p_{1}q_{0}\right)}$$

Among them, *α* is the type I error, *β* is the type II error, p_0_, and p_1_ represent the estimated exposure values of the control and exposed groups of the target population, respectively, q_1_ = (1 − p_1_), q_0_ = (1 − p_0_), and p_1_ = p_0_OR/ [1 + p_0_(OR − 1)] (13).

In a previous meta-analysis of the relationship between hypertension and cognitive decline, the prevalence of cognitive decline in hypertensive patients was 30% (14), so p_0_ = 0.30, then p_1_ = 0.46, with an expected OR = 2.0, *α* = 0.05, and *β* = 0.10. Substituting into the above formula, and since this study used 1:1 for matching, a minimum of 186 case groups and 186 control samples. Considering the follow-up miss rate, a total of 2,726 patients were finally included in this study.

### Diagnostic criteria

Hypertension is defined as a systolic blood pressure (SBP) of 140 mmHg or higher, a diastolic blood pressure (DBP) of 90 mmHg or higher, or the current use of antihypertensive medications (15). It is classified into three grades, Grade 1: SBP 140–159 mmHg and/or DBP 90–99 mmHg; Grade 2: SBP 160–179 mmHg and/or DBP 100–109 mmHg; Grade 3: SBP ≥ 180 mmHg and/or DBP ≥ 110 mmHg. Cognitive function was assessed using the Mini-Mental State Examination (MMSE), which evaluates five cognitive domains: orientation, attention, memory, language, and visuospatial abilities. The MMSE is a validated screening tool for cognitive decline and impairment that detects and differentiates between patients with cognitive decline and those with intact cognition (16, 17). The total score for the MMSE is 30, with higher scores reflecting better cognitive function. In this study, MMSE scores ranging from 18 to 27 were defined as early cognitive decline (18).

The WWI is determined by dividing the waist circumference (WC, measured in centimeters) by the square root of body weight (measured in kilograms) (10). Body weight and WC were assessed anthropometrically by trained healthcare professionals, whose proficiency was regularly verified. To ensure accuracy, subjects were advised to wear minimal clothing during weighing. Waist circumference was measured using a tape measured at specific anatomical landmarks.

### Ascertainment of covariates

Blood pressure was measured by a qualified nurse in a quiet environment. Additionally, fasting blood glucose (FBG), triglycerides (TG), total cholesterol (TC), high-density lipoproteins (HDL-C), low-density lipoproteins (LDL-C), and serum creatinine (Scr) levels were assessed in all subjects after an overnight fast.

Physical activity levels were measured using the Chinese version of the International Physical Activity Questionnaire Long Form (IPAQ-LC), which is reliable and shows adequate evidence of validity (19, 20).

### Statistical analysis

Continuous variables collected from participants were tested for normality according to the characteristics of the data; data conforming to a normal distribution were expressed as mean ± standard deviation (SD), and data not conforming to a normal distribution were expressed as median and interquartile range (IQR). Qualitative variables were expressed as relative numbers or percentages. WWI was transformed from a continuous variable to a categorical variable (quartile), and differences between subjects grouped by quartiles of WWI were compared in a multivariate logistic regression using quartile (Q1) as the reference group: Model 1 was not adjusted for any confounding variables and represented a univariate analysis. Model 2 was adjusted for sex, age, education level, marital status, type of work, smoking and drinking. The primary physiological and biochemical indicators (SBP, DBP, BMI, FBG, TG, TC, HDL-C, LDL-C, Scr) were incorporated into Model 2 to develop Model 3. Considering the impact of sleep disorders on HCD, Model 4 included the prevalence of sleep disorders in hypertensive patients. Data processing and analysis were performed using R version 4.4.0, along with Zstats 1.0.^1^

## Results

### Baseline clinical characteristics of subjects

Table 1 summarizes the baseline characteristics of hypertensive patients with normal cognitive function and those with cognitive decline. The mean age of the participants in this study was 72 years. The gender distribution was 44.7% male and 55.3% female. The risk of cognitive decline was higher among the older adults with hypertensive patients (*p* < 0.05).

**Table 1:** Baseline characteristics of hypertensive patients with normal cognitive function and cognitive decline.

Variables	Normal cognitive function (*n* = 1,363)	Cognitive decline (*n* = 1,363)	*p*
Age, years	72.72 ± 8.62	72.75 ± 8.68	0.926
Sex (Male), *n* (%)	615 (45.12)	604 (44.31)	0.672
Smoking, *n* (%)	153 (11.23)	132 (9.68)	0.189
Drinking, *n* (%)	178 (13.06)	150 (11.01)	0.099
MMSE score	29.31 ± 0.79	25.23 ± 2.34	< 0.001*
Marital status, *n* (%)			0.011*
Unmarried, divorced, or widowed	58 (4.26)	88 (6.46)	
Married	1,305 (95.74)	1,275 (93.54)	
Educational level, *n* (%)			< 0.001*
Illiteracy	132 (9.68)	188 (13.79)	
Primary school	588 (43.14)	711 (52.16)	
Middle school and above	643 (47.18)	464 (34.04)	
Type of work, *n* (%)			< 0.001*
Manual labor	912 (66.91)	1,071 (78.58)	
Mental labor	176 (12.91)	106 (7.78)	
Both manual and brain labor	275 (20.18)	186 (13.65)	
Physiological and biochemical indicators			
SBP, mmHg	147.67 ± 19.17	146.93 ± 20.12	0.326
DBP, mmHg	84.54 ± 12.12	84.72 ± 11.86	0.694
Body weight, kg	68.98 ± 9.72	67.53 ± 10.43	< 0.001*
WC, cm	87.73 ± 10.13	87.78 ± 9.61	0.885
BMI, kg/m^2^	25.34 ± 3.04	25.09 ± 3.27	0.044*
FBG, mmol/L	5.92 (5.26, 7.29)	5.91 (5.20, 7.20)	0.560
TG, mmol/L	1.27 (0.92, 1.82)	1.25 (0.89, 1.79)	0.292
TC, mmol/L	4.55 (3.79, 5.35)	4.40 (3.58, 5.28)	0.003*
HDL-C, mmol/L	1.21 (1.02, 1.45)	1.22 (0.99, 1.47)	0.995
LDL-C, mmol/L	2.72 (2.08, 3.42)	2.54 (1.90, 3.24)	< 0.001*
Scr, mg/dl	0.74 (0.63, 0.89)	0.75 (0.63, 0.88)	0.889
WWI index	10.54 (9.99, 11.10)	10.68 (10.21, 11.18)	0.001*
Sleep parameters			
PSQI score	6.00 (4.00, 8.00)	8.00 (6.00, 10.00)	< 0.001*
Sleep disorders, n (%)	478 (35.07)	700 (51.36)	< 0.001*
Physical activity level, *n* (%)			< 0.001*
Light	291 (21.35)	347 (25.46)	
Moderate	784 (57.52)	842 (61.78)	
Vigorous	288 (21.13)	174 (12.77)	

Data are presented as the mean ± SD, median [IQR], or n (%). MMSE, Mini-mental State Examination; WC, waist circumference; BMI, body mass index; FBG, fasting blood glucose; TG, triglyceride; TC, total cholesterol; HDL-C, High density lipoprotein cholesterol; LDL-C, low-density lipoprotein cholesterol; Scr, serum creatinine; WWI, weight-adjusted waist; PSQI, Pittsburgh sleep quality index. *Statistically significant (*p* < 0.05).

Table 2 categorizes the participants into quartiles based on their WWI values. Q1 corresponds to values ranging from 7.00 to 10.11, Q2 from 10.11 to 10.62, Q3 from 10.62 to 11.16, and Q4 from 11.16 to 16.76. The prevalence of cognitive decline significantly increased with higher WWI index among hypertensive patients (Q1: 41.75%; Q2: 50.07%; Q3: 54.37%; Q4: 53.64%, *p* < 0.001).

**Table 2:** Baseline characteristics of the study population based on the weight-adjusted waist index.

Variables	Q1 (7.00 ~ 10.11) (*n* = 673)	Q2 (10.11 ~ 10.62) (*n* = 681)	Q3 (10.62 ~ 11.16) (*n* = 686)	Q4 (11.16 ~ 16.76) (*n* = 686)	*p*
Age, years	73.27 ± 9.47	72.62 ± 8.57	71.81 ± 8.24	73.23 ± 8.19	0.005*
Sex (Male), *n* (%)					
	365 (54.23)	326 (47.87)	294 (42.86)	234 (34.11)	< 0.001*
Smoking, *n* (%)					
	94 (13.97)	64 (9.40)	76 (11.08)	51 (7.43)	< 0.001*
Drinking, *n* (%)					
	101 (15.01)	74 (10.87)	75 (10.93)	78 (11.37)	< 0.001*
MMSE score					
	27.78 ± 2.38	27.31 ± 2.66	27.02 ± 2.81	26.99 ± 2.79	< 0.001*
Cognitive decline, *n* (%)					
	281 (41.75)	341 (50.07)	373 (54.37)	368 (53.64)	< 0.001*
Marital status, *n* (%)					0.049*
Unmarried, divorced, or widowed					
	25 (3.71)	33 (4.85)	40 (5.83)	48 (7.00)	
Married					
	648 (96.29)	648 (95.15)	646 (94.17)	638 (93.00)	
Educational level, *n* (%)					< 0.001*
Illiteracy					
	69 (10.25)	80 (11.75)	78 (11.37)	93 (13.56)	
Primary school					
	272 (40.42)	335 (49.19)	351 (51.17)	341 (49.71)	
Middle school and above					
	332 (49.33)	266 (39.06)	257 (37.46)	252 (36.73)	
Type of work, *n* (%)					< 0.001*
Manual labor					
	420 (62.41)	512 (75.18)	539 (78.57)	512 (74.64)	
Mental labor					
	73 (10.85)	68 (9.99)	71 (10.35)	70 (10.20)	
Both manual and brain labor					
	180 (26.75)	101 (14.83)	76 (11.08)	104 (15.16)	
Physiological and biochemical indicators					
SBP, mmHg					
	146.56 ± 19.04	148.97 ± 20.22	147.63 ± 20.08	146.05 ± 19.14	0.032*
DBP, mmHg					
	84.18 ± 11.83	86.10 ± 11.58	85.06 ± 12.83	83.17 ± 11.50	< 0.001*
Body weight, kg					
	69.92 ± 9.84	68.94 ± 9.69	67.70 ± 9.25	66.51 ± 11.22	< 0.001*
WC, cm					
	79.04 ± 7.18	85.85 ± 6.19	89.21 ± 6.14	96.75 ± 10.01	< 0.001*
BMI, kg/m^2^					
	25.12 ± 3.08	25.23 ± 2.97	25.17 ± 2.89	25.34 ± 3.65	0.623
FBG, mmol/L					
	5.94 (5.20, 7.40)	5.93 (5.29, 7.21)	5.81 (5.25, 7.21)	5.98 (5.22, 7.19)	0.702
TG, mmol/L					
	1.24 (0.87, 1.72)	1.27 (0.91, 1.80)	1.25 (0.93, 1.87)	1.26 (0.93, 1.82)	0.296
TC, mmol/L					
	4.38 (3.59, 5.23)	4.56 (3.76, 5.32)	4.42 (3.67, 5.37)	4.54 (3.64, 5.38)	0.132
HDL-C, mmol/L					
	1.20 (1.00, 1.44)	1.22 (1.01, 1.46)	1.21 (1.00, 1.46)	1.23 (1.03, 1.47)	0.363
LDL-C, mmol/L					
	2.60 (2.04, 3.32)	2.67 (2.02, 3.39)	2.64 (2.02, 3.34)	2.58 (1.93, 3.34)	0.433
Scr, mg/dl					
	0.77 (0.64, 0.91)	0.74 (0.63, 0.88)	0.73 (0.62, 0.88)	0.73 (0.61, 0.86)	< 0.001*
Sleep parameters					
PSQI score					
	7.00 (5.00, 9.00)	7.00 (5.00, 9.00)	7.00 (5.00, 9.00)	7.00 (5.00, 9.00)	0.167
Sleep disorders, n (%)					
	294 (43.68)	277 (40.68)	314 (45.77)	293 (42.71)	< 0.001*
Physical activity level, *n* (%)					< 0.001*
Light	190 (28.23)	137 (20.12)	131 (19.10)	180 (26.24)	
Moderate	386 (57.36)	408 (59.91)	452 (65.89)	380 (55.39)	
Vigorous	97 (14.41)	136 (19.97)	103 (15.01)	126 (18.37)	

Data are presented as the mean ± SD, median [IQR], or n (%). MMSE, Mini-mental State Examination; WC, waist circumference; BMI, body mass index; FBG, fasting blood glucose; TG, triglyceride; TC, total cholesterol; HDL-C, High density lipoprotein cholesterol; LDL-C, low-density lipoprotein cholesterol; Scr, serum creatinine; WWI, weight-adjusted waist; PSQI, Pittsburgh sleep quality index. *Statistically significant (*p* < 0.05).

### Associations between MMSE scores and multidimensional factors

**Figure 1:**
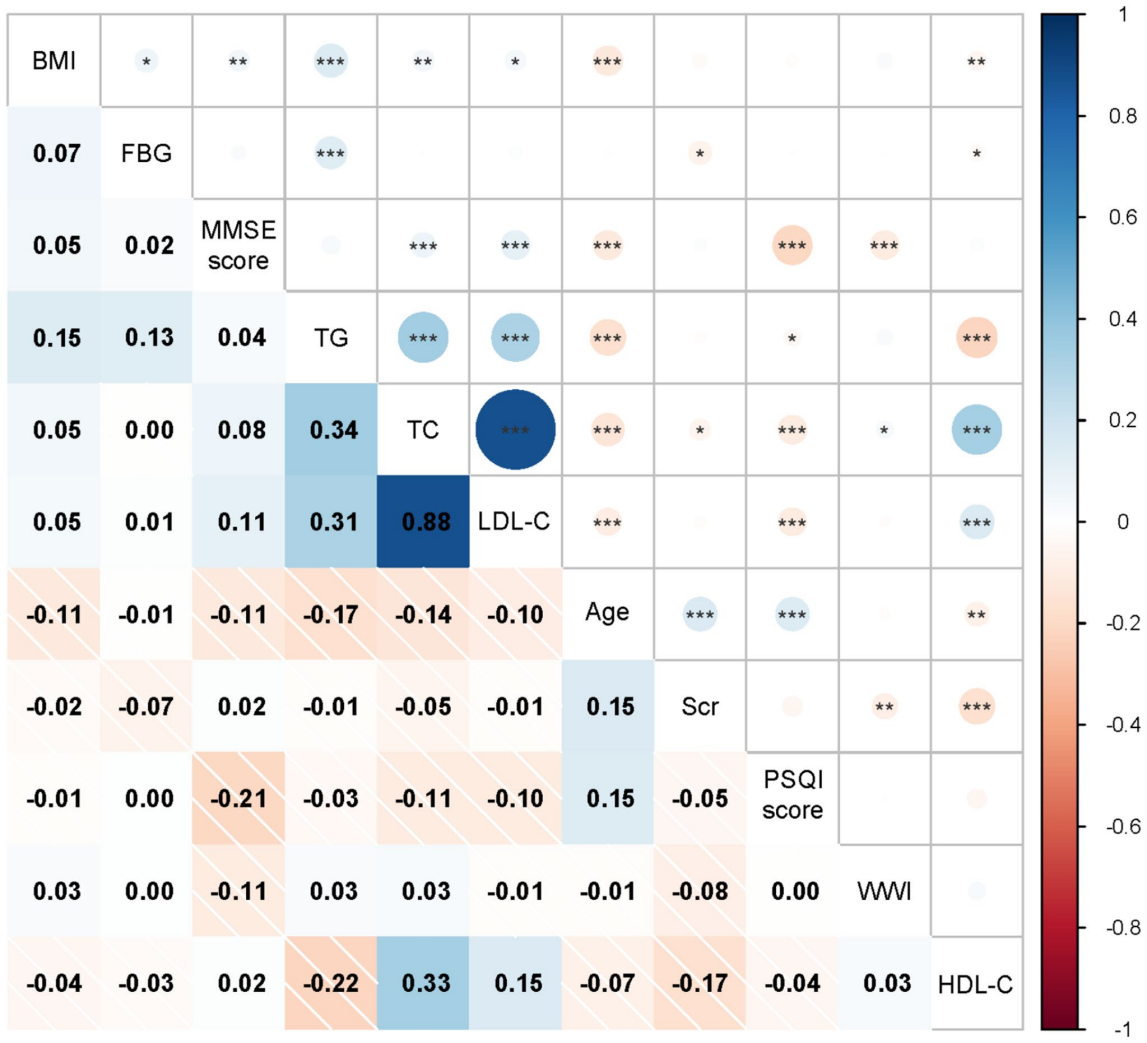
Associations between MMSE score and multidimensional factor, the x-axis and y-axis represent variable names, including BMI, FBG, MMSE score, TG, TC, LDL-C, Age, Scr, PSQI score, WWI, HDL-C. Each cell shows the correlation coefficient between the two variables, ranging from − 1 (dark red, indicating negative correlation) to 1 (dark blue, indicating positive correlation). *Indicates a significance level of *p* < 0.05, ** indicates a significance level of *p* < 0.01, *** indicates a significance level of *p* < 0.001.

Figure 1 shows a significant negative correlation between age, WWI, PSQI, and MMSE scores in this study (*p* < 0.001). Additionally, significant positive correlations were observed between TC, LDL-C and MMSE scores (*p* < 0.001). Although the correlations between these variables and MMSE scores were relatively weak, the findings suggest that increases in age, WWI, and PSQI scores may negatively impact cognitive functioning. On the contrary, higher TC and LDL-C levels were associated with better cognitive function. This finding seems to be contradictory because TC and LDL-C are usually associated with adverse cardiovascular outcomes. Previous studies have also obtained conflicting data on the relationship between blood lipids and cognitive function (21–24), suggesting that the effects of TC and LDL-C on cognitive function may be dual.

### Logistic regression analysis

Table 3 shows the relationship between WWI and HCD and the results of the multivariate logistic regression analysis. In the original model, there was a statistically significant positive correlation between WWI and HCD, with an OR of 1.13 (95% CI: 1.05 ~ 1.22; *p* < 0.001). In Model 2, after adjusting for factors such as age, sex, educational level, marital status, type of work, smoking and drinking, the positive association remained highly significant (OR = 1.11; 95% CI: 1.03 ~ 1.20; *p* < 0.001). In Model 3, even after adjusting for additional variables including BMI, FBG, TG, TC, HDL-C, LDL-C, and Scr, the correlation between WWI and HCD continued to be statistically significant (OR = 1.10; 95% CI: 1.02 ~ 1.19; *p* < 0.001). In Model 4, which was developed based on Model 3, this significant correlation persisted (OR = 1.12; 95% CI: 1.04 ~ 1.21; *p* < 0.001), even after accounting for sleep disorders. This indicates that the prevalence of cognitive decline among hypertensive patients increased by 12% with each unit increase in WWI. The statistical significance of the trend test showed that the strong correlation between WWI and cognitive decline in hypertensive patients persisted even after stratifying WWI into quartiles (*p* < 0.001). In Model 4, the prevalence of cognitive decline in hypertensive patients rose by 53% when comparing the highest quartile of WWI to the lowest quartile.

**Table 3:** Prevalence of HCD and 95% confidence intervals based on WWI.

Variables	Model1		Model2		Model3		Model4	
	OR (95%CI)	*p*	OR (95%CI)	*p*	OR (95%CI)	*p*	OR (95%CI)	*p*
WWI (continuous)	1.13 (1.05 ~ 1.22)	< 0.001*	1.11 (1.03 ~ 1.20)	< 0.001*	1.10 (1.02 ~ 1.19)	< 0.001*	1.12 (1.04 ~ 1.21)	< 0.001*
WWI quantile								
Q1 (7.00 ~ 10.11)	1.00(Reference)		1.00(Reference)		1.00(Reference)		1.00(Reference)	
Q2(10.11 ~ 10.62)	1.40 (1.13 ~ 1.73)	0.002*	1.28 (1.03 ~ 1.60)	0.027*	1.28 (1.02 ~ 1.59)	0.030*	1.32 (1.05 ~ 1.65)	0.030*
Q3 (10.62 ~ 11.16)	1.66 (1.34 ~ 2.06)	< 0.001*	1.51 (1.21 ~ 1.88)	< 0.001*	1.51 (1.21 ~ 1.89)	< 0.001*	1.52 (1.21 ~ 1.90)	< 0.001*
Q4 (11.16 ~ 16.76)	1.61 (1.30 ~ 2.00)	< 0.001*	1.50 (1.21 ~ 1.88)	< 0.001*	1.48 (1.18 ~ 1.85)	< 0.001*	1.53 (1.22 ~ 1.92)	< 0.001*

In multiple logistic regression analyses, WWI was converted from a continuous variable to a categorical variable (quartiles).

OR: odds ratio; 95% Cl: 95% confidence interval.

Model1: Crude.

Model2: Adjust: Age, Sex, Educational level, Marital status, Type of Work, Smoking, Drinking.

Model3: Adjust: Age, Sex, Educational level, Marital status, Type of Work, Smoking, Drinking, SBP, DBP, BMI, FBG, TG, TC, HDL-C, LDL-C, Scr.

Model4: Adjust: Age, Sex, Educational level, Marital status, Type of Work, Smoking, Drinking, SBP, DBP, BMI, FBG, TG, TC, HDL-C, LDL-C, Scr and sleep disorders.*Statistically significant (*p* < 0.05).

## Discussion

In this case-control study based on a large sample size, we examined the association between a new obesity index WWI and cognitive decline in hypertensive populations. Cognitive decline involves multiple factors (25, 26). Therefore, understanding the risk factors associated with HCD is essential for effective prevention and treatment strategies, and WWI shows promise as a new predictor in the diagnosis of HCD.

With the rising prevalence of obesity and obesity-related diseases worldwide (27), it is essential to accurately assess obesity and identify individuals at risk for HCD in clinical practice. Previous studies have reported that the relationship between cognitive decline and obesity-related parameters is controversial, that is, the ‘obesity paradox’. Some studies have shown that the lower BMI, the better the cognitive function, and the higher BMI, the worse the cognitive function (28, 29). However, some other studies have shown that a higher BMI prevents cognitive decline, while a lower BMI increases the likelihood of poor cognitive performance (30, 31). The reason may be that anthropometric indicators related to obesity (such as BMI) have inherent limitations due to the inability to distinguish between muscle mass and fat mass (8, 32, 33). To better explore the correlation between obesity and cognitive decline, recent studies have tended to use non-traditional obesity indicators to measure obesity and test the exact relationship between the two. A meta-analysis showed that subjects with a high triglyceride-glucose index (TyG) index were significantly associated with a higher risk of cognitive impairment compared to subjects with a low TyG index [RR: 1.39, 95% CI: 1.22 to 1.59, *p* < 0.001; I^2^ = 45%] (34). In addition, another clinical study showed that a higher lipid accumulation product (LAP) (OR = 1.037, 95% CI = 1.025–1.050, *p* < 0.01) was associated with a higher risk of mild cognitive impairment (MCI). After correcting for age, gender, lifestyle risk factors, duration of diabetes mellitus, LDL, HbA1c, education, insulin use, statin use, and diabetic peripheral neuropathy, a high LAP index was still associated with an increased risk of MCI (OR = 1.047, 95% CI = 1.031–1.063, *p* < 0.01) (35). However, most of these non-traditional obesity indicators are computationally complex and are poorly operationalized in practical applications.

The weight-adjusted waist index, as a newly developed obesity parameter, combines the advantages of WC while weakening the correlation with BMI compared to traditional formulas based on BMI, enabling the assessment of fat and muscle mass components independent of BMI. Previously, Kim et al.’s cross-sectional study of 602 participants aged 65 years in the Anshan Geriatric Study found that WWI was better able to differentiate between adiposity and muscle mass components compared with BMI (36). Therefore, WWI may be more reliable for detecting cognitive decline than commonly used body composition indices such as BMI. A previous cross-sectional study has reported that WWI is positively associated with dementia in a population of hypertensive patients (12), which is highly consistent with our findings. Although the interaction effects between several subgroups (such as education level, marital status, type of work, and physical activity) were not statistically significant in our study, these findings do not completely rule out the possibility of subgroup differences.

The mechanism by which WWI is positively associated with cognitive decline may be related to obesity, inflammatory responses, and metabolic abnormalities. WWI was positively correlated with the abdominal fat area and visceral fat area but negatively correlated with the abdominal muscle area, suggesting that an increase in WWI may reflect a state of excessive fat accumulation in the body as well as dysfunction of adipose tissue (8, 36, 37). Obesity is a chronic, persistent inflammatory state. It increases the production of various pro-inflammatory cytokines and adipocytokines, leading to an inflammatory response, endothelial dysfunction, and consequent adverse effects on cognitive function (38–41); At the same time, excess pro-inflammatory factors can lead to insulin resistance, which in turn triggers metabolic syndrome, which is associated with an increased risk of cognitive decline (42–44). Secondly, obesity-related inflammation can lead to leptin resistance and decreased adiponectin secretion, and induce cerebral neurodegeneration and neurodegenerative diseases (44–47). Finally, obesity harms cerebrovascular function and the blood–brain barrier, thus affecting cognitive ability (48, 49). In addition, increased age may also partially explain the mechanism of the association between WWI and cognitive decline. As individuals age, changes in body composition, including increased visceral fat and changes in adipose tissue distribution, can affect cognitive function (50). Future studies involving larger and more diverse populations may help clarify the role of these factors in moderating the relationship between WWI and HCD.

### Limitations and strengths

Our study is based on the hypertension follow-up system in Shandong Province, which is a hypertension sampling survey system in Shandong Province, following strict research programs and quality control measures. To enhance the reliability of our research results, we have adjusted multiple potential covariates to ensure the reliability of the results. Due to the simplicity and ease of calculation, WWI may become a practical tool for managing and intervening cognitive decline in patients with hypertension in clinical practice.

However, this study has several limitations. First, as a case-control study, it cannot establish a causal relationship between WWI and HCD. Second, although we adjusted to many important covariates, we could not completely exclude the effects of other potential confounders. While WWI predicted a decline in cognitive ability in our study, we recognized that other indicators derived from anthropometric indicators [e.g., TyG-body mass index (51)] may also complement cardiovascular risk prediction. Future studies should explore the combined utility of WWI and these indicators to better understand their influence on cognitive function in patients with hypertension.

Furthermore, we found that higher TC and LDL-C levels were associated with better cognitive performance. This finding seems contradictory, as high levels of these lipids are often associated with negative health outcomes, particularly cardiovascular risk (52, 53). However, combined with previous studies (21–24) and our findings, the relationship between TC, LDL-C, and cognitive function does not seem to be one-way but is influenced by a series of physiological and pathological factors. Therefore, the effect of blood lipid levels on cognitive function in patients with hypertension needs further study.

Finally, since the sample of this study is limited to individuals with hypertension in Shandong Province, the generalization of our results in the wider population of hypertension in China remains to be verified.

## Conclusion

Our study demonstrated that elevated levels of WWI in hypertensive patients were significantly associated with an increased risk of cognitive decline. This finding suggests that WWI may serve as a potential intervention indicator for mitigating the risk of cognitive decline in hypertensive individuals. However, further longitudinal studies are necessary to clarify the precise causality of this relationship.

## Data Availability

The data analyzed in this study is subject to the following licenses/restrictions: The datasets used and analysed during the current study available from the corresponding author on reasonable request. Requests to access these datasets should be directed to Huachen Jiao, liyixuan0531@163.com.

## Footnotes

^1^ www.zstats.net

## Glossary

HCD: cognitive decline in hypertension
WWI: waist circumference index
MMSE: Mini-mental State Examination
WC: waist circumference
BMI: body mass index
FBG: fasting blood glucose
TG: triglyceride
TC: total cholesterol
HDL-C: High density lipoprotein cholesterol
LDL-C: low-density lipoprotein cholesterol
Scr: serum creatinine
WWI: weight-adjusted waist
PSQI: Pittsburgh sleep quality index
ACEI: angiotensin-converting enzyme inhibitors
ARB: angiotensin receptor blocker
CCB: calcium channel blocker
IPAQ-L: International Physical Activity Questionnaire Long Form
TyG: triglyceride-glucose index
LAP: lipid accumulation product
MCI: mild cognitive impairment

## References

[1] Kim JS Sung Kim J Bin Bae J Won Han J Jong Oh D Wan Suh S. Association of estimated white matter hyperintensity age with cognition in elderly with controlled hypertension. Neuroimage Clin. (2023) 37:103323. doi: 10.1016/j.nicl.2023.103323, PMID: 36638599 PMC9860510

[2] Poster viewing Session II. J Cereb Blood Flow Metab. (2017) 37:169–239. doi: 10.1177/0271678X17695989, PMID: 28366134 PMC5441166

[3] Biernacki M Baranowska-Kuczko M Niklińska GN Skrzydlewska E. The FAAH inhibitor URB597 modulates lipid mediators in the brain of rats with spontaneous hypertension. Biomol Ther. (2020) 10:1022. doi: 10.3390/biom10071022, PMID: 32664225 PMC7407381

[4] Haring B Wu C Coker LH Seth A Snetselaar L Manson JAE. Hypertension, dietary sodium, and cognitive decline: results from the women’s health initiative memory study. Am J Hypertens. (2016) 29:202–16. doi: 10.1093/ajh/hpv081, PMID: 26137952 PMC4723668

[5] Su J Xiao X. Factors leading to the trajectory of cognitive decline in middle-aged and older adults using group-based trajectory modeling: a cohort study. Medicine (Baltimore). (2022) 101:e31817. doi: 10.1097/MD.0000000000031817, PMID: 36451491 PMC9704883

[6] Wang X Li T Li H Li D Wang X Zhao A. Association of dietary inflammatory potential with blood inflammation: the prospective markers on mild cognitive impairment. Nutrients. (2022) 14:2417. doi: 10.3390/nu14122417, PMID: 35745147 PMC9229190

[7] Gombojav B Yi S-W Sull JW Nam CM Ohrr H. Combined effects of cognitive impairment and hypertension on total mortality in elderly people: the kangwha cohort study. Gerontology. (2011) 57:490–6. doi: 10.1159/000323759, PMID: 21358170

[8] Xie F Xiao Y Li X Wu Y. Association between the weight-adjusted-waist index and abdominal aortic calcification in United States adults: results from the national health and nutrition examination survey 2013–2014. Front Cardiovasc Med. (2022) 9:948194. doi: 10.3389/fcvm.2022.948194, PMID: 36186965 PMC9515490

[9] Park Y Kim NH Kwon TY Kim SG. A novel adiposity index as an integrated predictor of cardiometabolic disease morbidity and mortality. Sci Rep. (2018) 8:16753. doi: 10.1038/s41598-018-35073-4, PMID: 30425288 PMC6233180

[10] Zhao P Shi W Shi Y Xiong Y Ding C Song X. Positive association between weight-adjusted-waist index and hyperuricemia in patients with hypertension: the China H-type hypertension registry study. Front Endocrinol. (2022) 13:1007557. doi: 10.3389/fendo.2022.1007557, PMID: 36277696 PMC9582276

[11] Li Q Qie R Qin P Zhang D Guo C Zhou Q. Association of weight-adjusted-waist index with incident hypertension: the rural Chinese cohort study. Nutr Metab Cardiovasc Dis. (2020) 30:1732–41. doi: 10.1016/j.numecd.2020.05.033, PMID: 32624344

[12] Zhou W Xie Y Yu L Yu C Bao H Cheng X. Positive association between weight-adjusted-waist index and dementia in the Chinese population with hypertension: a cross-sectional study. BMC Psychiatry. (2023) 23:519. doi: 10.1186/s12888-023-05027-w, PMID: 37468882 PMC10357774

[13] Wang R Zhao H Li B. Analytical clinical medicine research: key points of casecontrol study design. Shanghai Med. (2023) 44:23–5. doi: 10.3969/j.issn.1006-1533.2023.21.005

[14] Qin J He Z Wu L Wang W Lin Q Lin Y. Prevalence of mild cognitive impairment in patients with hypertension: a systematic review and meta-analysis. Hypertens Res. (2021) 44:1251–60. doi: 10.1038/s41440-021-00704-3, PMID: 34285378

[15] Joint Committee for Guideline Revision. 2018 chinese guidelines for prevention and treatment of hypertension-a report of the revision committee of chinese guidelines for prevention and treatment of hypertension. J geriatr cardiol: JGC. (2019) 16:182–241. doi: 10.11909/j.issn.1671-5411.2019.03.014 31080465 PMC6500570

[16] Croll PH Vinke EJ Armstrong NM Licher S Vernooij MW de Baatenburg Jong RJ. Hearing loss and cognitive decline in the general population: a prospective cohort study. J Neurol. (2020) 268:860. doi: 10.1007/s00415-020-10208-8, PMID: 32910252 PMC7914236

[17] Tombaugh TN McIntyre NJ. The mini-mental state examination: a comprehensive review. J Am Geriatr Soc. (1992) 40:922–35. doi: 10.1111/j.1532-5415.1992.tb01992.x, PMID: 1512391

[18] Zhong X Yu J Jiang F Chen H Wang Z Teng J. A risk prediction model based on machine learning for early cognitive impairment in hypertension: development and validation study. Front Public Health. (2023) 11:1143019. doi: 10.3389/fpubh.2023.1143019, PMID: 36969637 PMC10034177

[19] Xue B Xue Y Dong F Zheng X Shi L Xiao S. The impact of socioeconomic status and sleep quality on the prevalence of multimorbidity in older adults. Front Public Health. (2022) 10:959700. doi: 10.3389/fpubh.2022.959700, PMID: 36225792 PMC9548700

[20] Macfarlane D Chan A Cerin E. Examining the validity and reliability of the chinese version of the international physical activity questionnaire, long form (IPAQ-LC). Public Health Nutr. (2011) 14:443–50. doi: 10.1017/S1368980010002806, PMID: 20939939

[21] Zhou F Deng W Ding D Zhao Q Liang X Wang F. High low-density lipoprotein cholesterol inversely relates to dementia in community-dwelling older adults: the Shanghai aging study. Front Neurol. (2018) 9:952. doi: 10.3389/fneur.2018.00952, PMID: 30483213 PMC6240682

[22] Liu H Zou L Zhou R Zhang M Gu S Zheng J. Long-term increase in cholesterol is associated with better cognitive function: evidence from a longitudinal study. Front Aging Neurosci. (2021) 13:691423. doi: 10.3389/fnagi.2021.691423, PMID: 34220488 PMC8248815

[23] Stough C Pipingas A Camfield D Nolidin K Savage K Deleuil S. Increases in total cholesterol and low density lipoprotein associated with decreased cognitive performance in healthy elderly adults. Metab Brain Dis. (2019) 34:477–84. doi: 10.1007/s11011-018-0373-5, PMID: 30649667

[24] Guo Y Li P Ma X Huang X Liu Z Ren X. Association of Circulating Cholesterol Level with cognitive function and mild cognitive impairment in the elderly: a community-based population study. Curr Alzheimer Res. (2020) 17:556–65. doi: 10.2174/1567205017666200810165758, PMID: 32781960

[25] Zhang T Wu X Yuan H Huang S Park S. Mitigation of memory impairment with fermented fucoidan and λ-carrageenan supplementation through modulating the gut microbiota and their metagenome function in hippocampal amyloid-β infused rats. Cells. (2022) 11:2301. doi: 10.3390/cells11152301, PMID: 35892598 PMC9367263

[26] A Armstrong, R. Risk factors for Alzheimer’s disease. Folia Neuropathol. (2019) 57:87–105. doi: 10.5114/fn.2019.85929, PMID: 31556570

[27] Verkouter I de Mutsert R Smit RAJ Trompet S Rosendaal FR van Heemst D. The contribution of tissue-grouped BMI-associated gene sets to cardiometabolic-disease risk: a Mendelian randomization study. Int J Epidemiol. (2020) 49:1246. doi: 10.1093/ije/dyaa070, PMID: 32500151 PMC7660142

[28] Rubin LH Gustafson D Hawkins KL Zhang L Jacobson LP Becker JT. Midlife adiposity predicts cognitive decline in the prospective multicenter AIDS cohort study. Neurology. (2019) 93:e261–71. doi: 10.1212/WNL.0000000000007779, PMID: 31201294 PMC6656644

[29] Gong H-J Tang X Chai YH Qiao YS Xu H Patel I. Relationship between weight-change patterns and cognitive function: a retrospective study. JAD. (2023) 91:1085–95. doi: 10.3233/JAD-220788, PMID: 36565117

[30] Zhang W Chen Y Chen N. Body mass index and trajectories of the cognition among chinese middle and old-aged adults. BMC Geriatr. (2022) 22:613. doi: 10.1186/s12877-022-03301-2, PMID: 35870889 PMC9308927

[31] Aiken-Morgan AT Capuano AW Arvanitakis Z Barnes LL. Changes in body mass index are related to faster cognitive decline among african american older adults. J American Geriatrics Soc. (2020) 68:2662–7. doi: 10.1111/jgs.16814, PMID: 32978794 PMC8042656

[32] Komici K D’Amico F Verderosa S Piomboni I D’Addona C Picerno V. Impact of body composition parameters on lung function in athletes. Nutrients. (2022) 14:3844. doi: 10.3390/nu14183844, PMID: 36145219 PMC9500777

[33] Liu J Yang F Sun Q Gu T Yao J Zhang N. Fat mass is associated with subclinical left ventricular systolic dysfunction in patients with type 2 diabetes mellitus without established cardiovascular diseases. Diabetes Therapy. (2023) 14:1037. doi: 10.1007/s13300-023-01411-7, PMID: 37140878 PMC10203091

[34] Han Y Meng X Wang D. Association between triglyceride glucose index with cognitive impairment and dementia in adult population: a meta-analysis. Horm Metab Res. (2024) 56:737–48. doi: 10.1055/a-2284-5667, PMID: 38593823

[35] Yu Z-W Li X Wang Y Fu Y-H Gao X-Y. Association between lipid accumulation product and mild cognitive impairment in patients with type 2 diabetes. J Alzheimers Dis. (2020) 77:367–74. doi: 10.3233/JAD-200332, PMID: 32804130

[36] Kim NH Park Y Kim NH Kim SG. Weight-adjusted waist index reflects fat and muscle mass in the opposite direction in older adults. Age Ageing. (2021) 50:780–6. doi: 10.1093/ageing/afaa208, PMID: 33035293

[37] Kim JY Choi J Vella CA Criqui MH Allison MA Kim NH. Associations between weight-adjusted waist index and abdominal fat and muscle mass: multi-ethnic study of atherosclerosis. Diabetes Metab J. (2022) 46:747–55. doi: 10.4093/dmj.2021.0294, PMID: 35350091 PMC9532169

[38] Martinelli I Tayebati SK Roy P Micioni di Bonaventura MV Moruzzi M Cifani C. Obesity-related brain cholinergic system impairment in high-fat-diet-fed rats. Nutrients. (2022) 14:1243. doi: 10.3390/nu14061243, PMID: 35334899 PMC8948807

[39] Choi J Joseph L Pilote L. Obesity and C-reactive protein in various populations: a systematic review and meta-analysis. Obes Rev. (2013) 14:232–44. doi: 10.1111/obr.12003, PMID: 23171381

[40] Guo H Zhang Y Ma H Gong P Shi Y Zhao W. T-stage-specific abdominal visceral fat, haematological nutrition indicators and inflammation as prognostic factors in patients with clear renal cell carcinoma. Adipocytes. (2022) 11:133. doi: 10.1080/21623945.2022.2048546, PMID: 35285399 PMC8920171

[41] Shi S Ni L Tian Y Zhang B Xiao J Xu W. Association of obesity indices with diabetic kidney disease and diabetic retinopathy in type 2 diabetes: a real-world study. J Diabetes Res. (2023) 2023:1–11. doi: 10.1155/2023/3819830, PMID: 37096235 PMC10122582

[42] Lindberger E Wikström AK Bergman E Eurenius K Mulic-Lutvica A Sundström Poromaa I. Association of maternal central adiposity measured by ultrasound in early mid pregnancy with infant birth size. Sci Rep. (2020) 10:19702. doi: 10.1038/s41598-020-76741-8, PMID: 33184361 PMC7665175

[43] Hung C-C Cheng Y-W Chen W-L Fang W-H. Negative association between acrylamide exposure and metabolic syndrome markers in adult population. Int J Environ Res Public Health. (2021) 18:11949. doi: 10.3390/ijerph182211949, PMID: 34831705 PMC8624217

[44] Wang X-L Feng H-L Xu X-Z Liu J Han X. Relationship between cognitive function and weight-adjusted waist index in people ≥ 60 years old in NHANES 2011-2014. Aging Clin Exp Res. (2024) 36:30. doi: 10.1007/s40520-023-02649-8, PMID: 38334839 PMC10857983

[45] Chen W-D Lai LJ Lee KL Chen TJ Liu CY Yang YH. Is obesity a risk or protective factor for open-angle glaucoma in adults? A two-database, asian, matched-cohort study. J Clin Med. (2021) 10:4021. doi: 10.3390/jcm10174021, PMID: 34501469 PMC8432455

[46] Flores-Cordero JA Pérez-Pérez A Jiménez-Cortegana C Alba G Flores-Barragán A Sánchez-Margalet V. Obesity as a risk factor for dementia and alzheimer’s disease: the role of leptin. Int J Mol Sci. (2022) 23:5202. doi: 10.3390/ijms23095202, PMID: 35563589 PMC9099768

[47] Forny-Germano L De Felice FG Vieira MN d N. The role of leptin and adiponectin in obesity-associated cognitive decline and Alzheimer’s disease. Front Neurosci. (2018) 12:1027. doi: 10.3389/fnins.2018.01027, PMID: 30692905 PMC6340072

[48] Rask L Bendix L Harbo M Fagerlund B Mortensen EL Lauritzen MJ. Cognitive change during the life course and leukocyte telomere length in late middle-aged men. Front Aging Neurosci. (2016) 8:300. doi: 10.3389/fnagi.2016.00300, PMID: 28018213 PMC5145851

[49] Nguyen JCD Killcross AS Jenkins TA. Obesity and cognitive decline: role of inflammation and vascular changes. Front Neurosci. (2014) 8:375. doi: 10.3389/fnins.2014.00375, PMID: 25477778 PMC4237034

[50] Brinkley TE Leng I Register TC Neth BJ Zetterberg H Blennow K. Changes in adiposity and cerebrospinal fluid biomarkers following a modified mediterranean ketogenic diet in older adults at risk for alzheimer’s disease. Front Neurosci. (2022) 16:906539. doi: 10.3389/fnins.2022.906539, PMID: 35720727 PMC9202553

[51] Sodero G Rigante D Pane LC Sessa L Quarta L Candelli M. Cardiometabolic risk assessment in a cohort of children and adolescents diagnosed with hyperinsulinemia. Diseases. (2024) 12:119. doi: 10.3390/diseases12060119, PMID: 38920551 PMC11202913

[52] Montalvan Sanchez EE Urrutia SA Rodriguez AA Duarte G Murillo A Rivera R. Cardiovascular risk assessment in the resource limited setting of Western Honduras: an epidemiological perspective. Int J Cardiol Heart Vasc. (2020) 27:100476. doi: 10.1016/j.ijcha.2020.100476, PMID: 32309530 PMC7154315

[53] Yu S Wu X Ferguson M Simmen RC Cleves MA Simmen FA. Diets Containing Shiitake Mushroom Reduce Serum Lipids and Serum Lipophilic Antioxidant Capacity in Rats123. J Nutr. (2016) 146:2491–2496. doi: 10.3945/jn.116.239806 27798348 PMC5118771

